# Delirium After Mechanical Ventilation in Intensive Care Units: The Cognitive and Psychosocial Assessment (CAPA) Study Protocol

**DOI:** 10.2196/resprot.6660

**Published:** 2017-02-28

**Authors:** Daniella Bulic, Michael Bennett, Helen Rodgers, Mary Nourse, Patrick Rubie, Jeffrey CL Looi, Frank Van Haren

**Affiliations:** ^1^ University of New South Wales Faculty of Medicine University of New South Wales Randwick Australia; ^2^ Prince of Wales Hospital Anaesthetic Department Prince of Wales Hospital Randwick Australia; ^3^ Canberra Hospital Intensive Care Research Department Canberra Hospital Garran Australia; ^4^ Academic Unit of Psychiatry and Addiction Medicine Medical School Australian National University Canberra Australia; ^5^ Faculty of Medicine Melbourne Neuropsychiatry Centre, Department of Psychiatry, Faculty of Medicine, Dentistry and Health Sciences University of Melbourne Melbourne Australia; ^6^ Medical School College of Medicine, Biology & Environment Australian National University Canberra Australia

**Keywords:** intensive care, delirium, mechanical ventilation, cognition, psychosocial outcomes

## Abstract

**Background:**

In the intensive care unit (ICU), critical illness delirium occurs in the context of multiple comorbidities, multi-organ failure, and invasive management techniques, such as mechanical ventilation, sedation, and lack of sleep. Delirium is characterized by an acute confusional state defined by fluctuating mental status, inattention, and either disorganized thinking or an altered level of consciousness. The long-term cognitive and psychosocial function of patients that experience delirium in the ICU is of crucial interest because preliminary data suggest a strong association between ICU-related delirium and long-term cognitive impairment.

**Objective:**

The aim of this study is to explore the relationship between delirium in the ICU and adverse outcomes by following mechanically ventilated patients for one year following their discharge from the ICU and collecting data on their long-term cognition and psychosocial function.

**Methods:**

This study will be conducted by enrolling patients in two tertiary ICUs in Australia. We aim to recruit 200 patients who have been mechanically ventilated for more than 24 hours. Data will be collected at the following three time points: (1) at discharge where they will be administered the Mini-Mental State Examination (MMSE); (2) at 6 months after discharge from the ICU discharge where the Impact of Events Scale Revised (IES-R) and the Telephone Inventory for Cognitive Status (TICS) tests will be administered; and (3) at 12 months after discharge from the ICU where the patients will be administered the TICS and IES-R tests, as well as the Informant Questionnaire for Cognitive Decline in the Elderly (IQCODE). The IQCODE will be administered to their “person responsible” or the significant other of the patient.

**Results:**

Long-term cognition and psychosocial function will be the primary outcome of this study. Mortality will also be investigated as a secondary outcome. Active enrollment will take place until the end of September 2016 and data collection will conclude at the end of September 2017. The analysis and results are expected to be available by March 2018.

**Conclusion:**

Delirium during mechanical ventilation has been linked to longer ICU and hospital stays, higher financial burdens, increased risks of long-term cognitive impairment (ie, dementia), poor functional outcomes and quality of life, and decreased survival. However, delirium during mechanical ventilation in the ICU is not well understood. This study will advance our knowledge of the comprehensive, long-term effects of delirium on cognitive and psychosocial function.

**Trial Registration:**

Australian New Zealand Clinical Trials Registry (ANZCTR): ACTRN12616001116415; https://www.anzctr.org.au/Trial/Registration/TrialReview.aspx?id=371216 (Archived by WebCite at http://www.webcitation.org/ 6nfDkGTcW)

## Introduction

Delirium is a disturbance of consciousness, developed over a short period of time, where inattention is accompanied by a change in cognition and/or perceptual disturbance [1]. Delirium occurs in a variety of health care settings [2] and affects between 15% to 20% of general hospital patients [3,4], including as many as 80% of critically ill, intensive care unit (ICU) patients receiving mechanical ventilation [5].

In the ICU, delirium is associated with critical illness itself, particularly with multiple comorbidities and multi-organ failure, as well as management-related factors such as mechanical ventilation, sedation, and lack of sleep [2,6,7]. Delirium is also associated with adverse outcomes including death and long-term cognitive impairments [7-9], and potentially traumatic stress symptomatology. Several studies suggest delirium-related risks are cumulative and may foster the development of cognitive dysfunction, poorer functional status, and impair quality of life [7-23]. Despite literature reports of a reduced quality of life for survivors of critical illness and delirium in the ICU, the long-term follow-up of cognitive and psychosocial function still remains relatively unexplored. This study will address this by following up with patients for one year after discharge from the ICU. We will employ a set of tests that complement each other [24] in order to create a comprehensive view of a patient's cognition and psychosocial well-being at 12 months after leaving the ICU. These tests assess cognition and evidence of post-traumatic stress disorder (PTSD) [25], while also using each patients “person responsible” to assess their relative's and/or friend’s psychosocial function at 12 months after their discharge from the ICU in comparison to their pre-ICU psychosocial abilities. This study is expected to provide novel insight into the cognitive and psychosocial impact of ICU mechanical ventilation-related delirium and to assist in improving the care of critically ill intensive care patients by highlighting the importance of the need to monitor for the development of delirium.

## Methods

We are conducting a prospective case-control study in two ICUs in Australia: the Canberra Hospital in the Australian Capital Territory and the Prince of Wales Hospital in Sydney, New South Wales. The study was approved by the ACT Health Human Research Ethics Committee (ETH.6.12.130) and the Southern Health Human Research Committee (12/242 (HREC/I2/POWH/460). Since eligible participants are not able to give informed consent on enrolment due to their health status (ICU treatment), their substitute decision maker or “person responsible” will be identified. This person is approached to provisionally consent to participation on behalf of the patient. The “person responsible” is given a full explanation of the study and is provided with the Cognitive and Psychosocial Assessment (CAPA) study information sheet. They are then asked to consent to participate in the study on behalf of themselves and the patient. If the patient is not sedated or delirious, the study is explained to them; however, the “person responsible” is still required to complete and sign the consent form on behalf of themselves and the patient whilst the patient is ventilated. Further, consent from the “person responsible” authorizes their later involved in the assessment of patients’ overall function at the 12-month follow-up.

Despite being recruited to the study through their “person responsible,” the patients receive no other intervention other than standard protocol care during their ICU stay. The standard care in both recruiting sites involves administering the following during mechanical ventilation: (1) the Richmond Agitation and Sedation Score (RASS) [26,27], and (2) the Confusion Assessment Method for the ICU (CAM-ICU) [28].

After being recruited to the study, the patients have their sedation level assessed by the RASS [26,27] every 4 hours. The RASS is a scale that indicates the patient’s level of sedation and ranges from +4 to –5. A RASS score of 0 represents a patient who is alert and calm, while positive scores indicate different levels of agitation from restless (+1) to combative (+4). Negative scores indicate different levels of drowsiness from drowsy (–1) to unarousable (–5).

Patients with a RASS score between –2 and +3 will be administered the CAM-ICU [28]. The CAM-ICU is an adaptation of the Confusion Assessment Method and is widely used for diagnosing delirium with yes and no questions for use with non-speaking, mechanically ventilated patients in the ICU. The study participants are not exposed to any additional testing during their time in the ICU.

**Figure 1:**
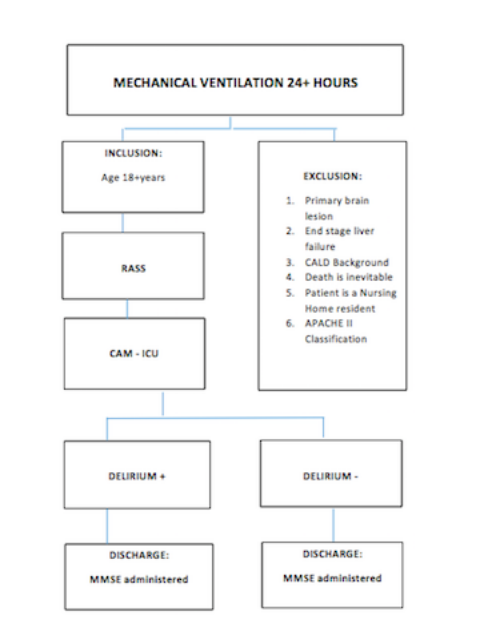
a. CALD Culturally and Linguistically Diverse Background; b. APACHE II Acute Physiology and Chronic Health Enquiry II; c. RASS Richmond Agitation Sedation Scale; d. CAM-ICU Confusion Assessment Method for the Intensive Care Unit; e. MMSE Mini Mental State Examination.

Patients assessed as suffering delirium via the CAM-ICU at any time are allocated into the delirium positive study group, while delirium-negative patients are re-tested on a daily basis during the time they remain mechanically ventilated. Those patients who never test positive for delirium are allocated to the delirium-negative group (Figure 1).

### Participants

Patients participating in this study are critically ill, mechanically ventilated, and may require immediate administration of sedative medications by infusion. As a consequence of the immediacy of the situation and the urgent need for sedation, we obtain provisional consent from the patient’s “person responsible” prior to enrolling patients in the study. Adult patients greater than 18 years of age are approached for recruitment after being mechanically ventilated for more than 24 hours. The exclusion criteria are (1) suspected acute primary brain lesion; (2) end stage liver failure or acute hepatic failure; (3) culturally and linguistically diverse (CALD) background—literature suggests that in times of crisis people automatically revert to their own language and our psychosocial questionnaires are not specifically designed for CALD [29,30]; (4) death is deemed imminent and inevitable; (5) patient is a nursing home resident and/or physical/cognitive decline is evident [31,32]; and (6) the Acute Physiology and Chronic Evaluation (APACHE II) [33] classification indicates underlying terminal illness.

### Procedure and Enrolment

**Figure 2:**
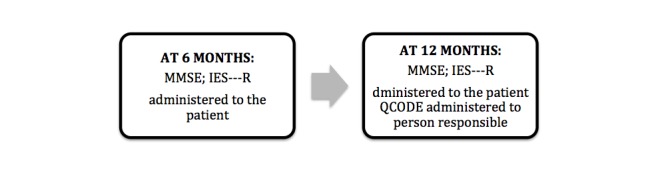
Assessments after hospital discharge. TICS: Telephone Interview for Cognitive Decline; IES-R: Impact of Events Scale Revised; IQCODE: Informant Questionnaire on Cognitive Decline in the Elderly.

Patients are asked to sign formal consent to participate upon discharge from the ICU. If they confirm their enrolment, they are administered the MMSE [34], the first cognitive assessment (Multimedia Appendix 1). At 6 months, the patients are contacted and administered the telephone-modified version of the MMSE called the Telephone Interview for Cognitive Status (TICS) [35,36] (Multimedia Appendix 2). They also receive the Impact of Events-Revised (IES-R) questionnaire [37,38] (Multimedia Appendix 3) by mail to assess for early symptoms of PTSD [25]. At 12 months after discharge, the patients are contacted, the TICS is administered, and they are mailed the IES-R. The Informant Questionnaire on Cognitive Decline (IQCODE) [37] (Multimedia Appendix 4) is also mailed to their “person responsible” who was identified at the time study recruitment. The hospital outcome assessments are recorded up to one year after discharge from the ICU (Figure 2).

For a pilot study that we conducted in 2011, we followed 8 patients (4 delirium-positive and 4 delirium-negative) for 3 months after their discharge from the ICU. During this time, the design and choice of measurement tools for this investigation were informed.

The questionnaires we employ in this study are well-established and validated in measuring cognition and psychosocial well-being. The MMSE, the most commonly used measure of cognitive function in hospitalized patients [34], is scored from 0 to 30, with lower scores indicating poorer performance. We administer the MMSE for the first time after patients formally consent and at the time of their discharge from the ICU. The TICS is a modified and validated version of MMSE and is administered to the patient over the phone at the 6- and 12-month follow-ups.

The IES-R is a non-diagnostic, self-report measure designed to assess subjective distress for any specific life event. The IES-R consists of the three subscales: hyper arousal, intrusion, and avoidance. These subscales parallel the Diagnostic and Statistical Manual of Mental Disorders IV (DSM-IV) criteria for PTSD. This questionnaire is administered to the patient at the 6- and 12-month follow-ups.

The IQCODE is a tool used to assess cognitive impairment in older people. It is widely used in conjunction with other cognitive tests and with no age limitations (personal communication DB and JL, August 2011). The IQCODE was found to correlate highly with conventional cognitive screening tests [25], such as the MMSE, and moderately with a range of neuropsychological tests, such as the Wechsler Adult Memory Scale (WAMS) and the Wechsler Adult Intelligence Scale (WAIS). Since the IQCODE provides information complementary to other brief cognitive tests, such as the MMSE [38], we supplemented the MMSE with the IQCODE to improve the study’s cognition screening accuracy, as well as gain a retrospective assessment of the patient's cognitive function.

We have obtained permission to use the above tools through direct contact with authors (Multimedia Appendix 5), and email correspondence (DB with DSW and AFJ, August 2011). All of the questionnaires have been validated, are found to be reliable and specific to psychosocial assessment [34-39], and are less time-consuming than other conventional cognitive and neuropsychological tests.

### Measures

This study aims to test the hypothesis that delirium during mechanical ventilation in the ICU promotes long-term cognitive and psychosocial decline and impacts one’s health-related quality of life when compared with non-delirium patients [8-23]. The data recorded at study enrolment, during their stay in the ICU, and at discharge are shown in Textbox 1.

**Data recorded at the time of patient enrolment in the study, during their stay in the intensive care unit, and at discharge**

Collected data

- At enrolment
  - Demographic data
    - Name
    - Mailing address
    - Contact number
    - Date of birth
    - Age
    - Indigenous status
    - Sex
  - Date and time of admission to the intensive care unit (ICU)
  - ICU admission diagnosis—Acute Physiology and Chronic Evaluation (APACHE II)
  - Date and time of first intubation
- Daily
  - Richmond Assessment Sedation Scale (RASS)
  - Confusion Assessment Method for the ICU (CAM-ICU)
- At discharge from the ICU
  - Delirium positive or negative
  - Date and time of discharge from the ICU
  - Survival status at discharge from the ICU
  - Mini-Mental State Examination (MMSE)
- After discharge from the ICU (episode outcome at 6 and 12 months)
  - Survival status
  - Dependency status
  - Cognitive status

### Sample Size

The primary outcome of this study is the relative decrement in MMSE at 6 months after discharge from the ICU. We used the most relevant clinical trial employing MMSE to quantify cognitive function of both delirium-positive and delirium-negative patients to estimate the expected outcomes for our patients at 6 months after discharge from the ICU in order to make a sample size calculation. We estimated the clinically important reductions expected in MMSE and calculated that, at a type 1 error rate of 5% (alpha .05), we can find the clinically significant difference of 2 points on the MMSE between groups with 80% power if we include a minimum of 81 patients in each group (delirious and not delirious). To allow for potential dropouts following recruitment, we plan to enroll 200 patients.

### Safety Monitoring

Both internal and external monitoring is utilized through this study. The principal investigator has on-site assessment through monthly meetings with the supervisors at both hospitals. An external person is also appointed at each research site to independently monitor the research process.

### Statistical Analysis

All statistical analysis will be done using Statistical Package for the Social Sciences (SPSS) Research Engine, Version 22.0 IBM SPSS Statistics (2015). We plan to employ the Student *t* test to compare mean outcomes in each group when assuming a normal underlying distribution can be justified and the Mann-Whitney U test to compare medians when this assumption fails. We will compare differences in proportionate outcomes using a Chi-square analysis. When calculating multiple time points for the purpose of comparing groups over time, we will apply appropriate correction for multiple testing using the Bonferroni correction. For all of these methods, we will determine if there is a statistically significant difference between the groups (*P* values less than .05). Results will be presented as differences between groups with 95% confidence intervals. We will also consider both analysis of covariance (parametric) or Kruskal-Wallis (non-parametric) testing as appropriate and will investigate the influence of potential confounders and correlations by logistic or multiple regression techniques. Further, we will randomly select two groups of patients (30 delirium-positive and 30 delirium-negative) to cross validate our results and assess whether they are stable for both samples.

## Results

The primary outcome of this study is the assessment of long-term cognition and psychosocial function. Patient mortality will be the study's secondary outcome. Active enrollment will take place until the end of September 2016 and data collection will conclude at the end of September 2017. The analysis and results are expected to be available by March 2018.

## Discussion

The use of sedation and multiple psychoactive medications in the ICU, combined with patients’ metabolic disturbances, underlying infections, and multi-organ failure, may promote delirium and create new or exacerbate existing cognitive impairments [10-23]. Burgeoning evidence has emerged in support of the association between the ICU experience of delirium and adverse patient outcomes, including longer hospital stays and poor functional recovery [40-43]. We hope this study, with its design and comprehensive data collection, will deliver novel insights into the impact of intensive care delirium on patients’ long-term cognitive and psychosocial outcomes, further informing the care of critically ill ICU patients.

## Appendix

Multimedia Appendix 1 Mini-Mental State Examination.

Multimedia Appendix 2 Telephone Interview for Cognitive Status (TICS).

Multimedia Appendix 3 Impact of Events Scale-Revised (IES-R).

Multimedia Appendix 4 Informant Questionnaire on Cognitive Decline in the Elderly (IQCODE).

Multimedia Appendix 5 MMSE:TICS permission.

## Abbreviations

APACHE II: Acute Physiology and Chronic Evaluation
CALD: culturally and linguistically diverse background
CAM-ICU: Confusion Assessment Method for ICU
ICU: intensive care unit
IES-R: Impact of Events Scale Revised
IQCODE: Informant Questionnaire on Cognitive Decline in the Elderly
MMSE: Mini-Mental State Examination
PTSD: post-traumatic stress disorder
TICS: Telephone Interview for Cognitive Status
