# Supplementary material for: Delirium After Mechanical Ventilation in Intensive Care Units: The Cognitive and Psychosocial Assessment (CAPA) Study Protocol
Source: JMIR Res Protoc. 2017 Feb 28;6(2):e31. doi: 10.2196/resprot.6660 (PMC5426842; doi:10.2196/resprot.6660)
Supplement: Multimedia Appendix 1 [file resprot_v6i2e31_app1.pdf]

Appendix 1

Mini-Mental State Examination (MMSE)

* To be administered at ICU discharge

Patient ID:______________________ Date:______________________

Instructions: Score one point for each correct response within each question or activity.

Maximum Score	Patient's Score	Questions
5		"What is the year? Season? Date? Day? Month?"
5		"Where are we now? State? County? Town/city? Hospital? Floor?"
3		The examiner names three unrelated objects clearly and slowly, then the instructor asks the patient to name all three of them. The patient's response is used for scoring. The examiner repeats them until patient learns all of them, if possible.
5		"I would like you to count backward from 100 by sevens." (93, 86, 79, 72, 65, …) Alternative: "Spell WORLD backwards." (D-L-R-O-W)
3		"Earlier I told you the names of three things. Can you tell me what those were?"
2		Show the patient two simple objects, such as a wrist watch and a pencil, and ask the patient to name them.
1		"Repeat the phrase: 'No ifs, ands, or buts.'"
3		"Take the paper in your right hand, fold it in half, and put it on the floor." (The examiner gives the patient a piece of blank paper.)
1		"Please read this and do what it says." (Written instruction is "Close your eyes.")
1		"Make up and write a sentence about anything." (This sentence must contain a noun and a verb.)
1		"Please copy this picture." (The examiner gives the patient a blank piece of paper and asks him/her to draw the symbol below. All 10 angles must be present and two must intersect.) 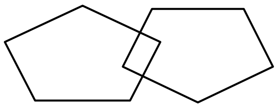
30		TOTAL
