# Supplementary material for: Delirium After Mechanical Ventilation in Intensive Care Units: The Cognitive and Psychosocial Assessment (CAPA) Study Protocol
Source: JMIR Res Protoc. 2017 Feb 28;6(2):e31. doi: 10.2196/resprot.6660 (PMC5426842; doi:10.2196/resprot.6660)
Supplement: Multimedia Appendix 2 [file resprot_v6i2e31_app2.pdf]

TICS™ Telephone Interview for Cognitive Status™

by Jason Brandt, PhD, and Marshal F. Folstein, MD

Record Form

Examinee Name or ID ______________________ Date of Testing ___/___/___ Date of Birth ___/___/___ Age ______

Address of Examinee ____
Street City

_____________________________________ *Education Level (no. of years or degree)* ______________
State Zip Code

Name of Examiner ___________________________________ Name of Proctor ______________________

Suggested Qualitative Interpretive Ranges for TICS Total Score Range

- ☐ 33-41 Nonimpaired
- ☐ 26-32 Ambiguous
- ☐ 21-25 Mildly Impaired
- ☐ ≤ 20 Moderately to Severely Impaired

TICS *T* scores

For individuals with < 12 years of education and ≥ 65 years of age	For individuals with ≥ 12 years of education and ≥ 60 years of age
MMSE Total score equivalent of TICS Total score = ________<br>(Obtain from Table 3 of TICS Professional Manual)<br><br>*T* score = ________<br>(Obtain from Tables A10-A14 of MMSE Clinical Guide)	*T* score = ________<br>(Obtain from Table 2 of TICS Professional Manual)<br><br>90% Confidence Interval ________ to ________<br>(*T* score – 8) (*T* score + 8)

PAR® • 16204 N. Florida Ave. • Lutz, FL 33549 • 1.800.331.8378 • www.parinc.com

Copyright © 1987, 2003 by PAR. All rights reserved. May not be reproduced in whole or in part in any form or by any means without written permission of PAR. This form is printed in blue and black ink on white paper. Any other version is unauthorized.

9 8 7 6 5 Reorder #RO-5182 Printed in the U.S.A.

WARNING! PHOTOCOPYING OR DUPLICATION OF THIS FORM WITHOUT PERMISSION IS A VIOLATION OF COPYRIGHT LAWS.

TICS Administration Instructions

Prior to the TICS administration, examiners should ensure that they have obtained all of the necessary information (e.g., examinee's full name, complete address, etc.).

Instructions to Proctor

In a couple of minutes, I am going to be asking [*examinee's name*] a number of different questions to test [*his/her*] thinking and memory. Before we start, I need to ask you whether the address I have for your current location is correct. Please don't repeat it out loud if [*examinee's name*] is in the room with you, since I will be asking [*him/her*] the same question in a few minutes. Is your current address [*examinee's address*]? If the answer is "no," please ask the proctor either to step into another room before giving you the correct address or to have the examinee leave the room briefly before giving you the address. Then go on to say, **Please be sure that all papers, pencils, books, calendars, newspapers, and everything else that might provide distraction or visual cues are removed from [*examinee's name*] sight. Also, please be sure that the room is quiet; there should be no television, radio, or music playing.**

Some of the questions may be difficult for [*examinee's name*] to answer. [*He/She*] may ask you for help. If [*he/she*] does, just encourage [*him/her*] to do as well as [*he/she*] can. [*He/She*] should guess if necessary. Please do not give [*him/her*] any answers or hints. O.K.? If you and [*examinee's name*] are ready, please put [*him/her*] on the phone.

Instructions to Examinee

I am going to ask you some questions to test your memory. Some of these are likely to be easy for you, but some may be difficult. Please bear with me and try to answer all the questions as best you can. If you can't answer a question, don't worry. Just try your best. Are you ready? These instructions may be repeated *verbatim* or paraphrased, if necessary. For each of the actual TICS items, except Item 5 and Item 8, single repetitions are permitted.

Item	Item response	Scoring criteria	Max. score	Item score
1. **Please tell me your full name.**		1 point for correct first name (or nickname) and 1 point for correct last name	2	
2. **What is today's date?** Probe for month, date, year, day of week, and season if any not provided spontaneously (e.g., **What day of the week is it?** *or* **What season is it?**).		1 point each for precisely correct month, date, year, day of the week, and season (e.g., a hot day in early June is *not* summer)	5	
3. **Where are you right now?** Probe for house number, street, city, state, and zip code if any not provided spontaneously (e.g., **What number is that? What is your zip code?**).		1 point each for correct house number, street, city, state, and zip code (5-digit zip code is sufficient). If examinee is in a facility with no house number (e.g., hospital, nursing home), the name of the facility may be substituted for the house number.	5	
4. **Please count backward from 20 to 1.** If examinee makes an error, ask him or her to try again.		2 points if completely correct on first trial<br>1 point if completely correct on second trial	2	
5. **I am going to read you a list of 10 words. Please listen carefully and try to remember them. When I am done, tell me as many of the words as you can, in any order. Ready? The words are** (*pause*) **cabin, pipe, elephant, chest, silk, theater, watch, whip, pillow, giant.** (*Pause.*) **Now tell me all the words you can remember.** The words should be read at approximately one word every 2 seconds. No repetitions of the word list are permitted.		1 point for each correctly recalled word<br>0 points for incorrect responses, repetitions, or intrusions	10	

Item	Item response	Scoring criteria	Max. score	Item score
6. **I would like you to take the number 100 and subtract 7.** (Pause for a response.) **Now keep subtracting 7 from the answer until I tell you to stop.** No further prompts or instructions are given, except to **"keep going."** Stop the examinee after five serial subtractions.		1 point for each correct subtraction<br>Do not inform examinee of incorrect responses, but allow subtractions to be made from the last response.<br>For example, "93, 85, 78, 71, 65" would be awarded 3 points.	5	
7. **What do people usually use to cut paper?** (Pause for a response.)<br>**How many things are in a dozen?** (Pause for a response.)<br>**What do you call the prickly green plant that lives in the desert?** (Pause for a response.)<br>**What animal does wool come from?**		1 point each for "scissors" or "shears"<br>1 point for "12"<br>1 point for "cactus"<br>1 point for "sheep" or "lamb"	4	
8. **Please repeat this after me: "No ifs, ands, or buts."** (Pause for a response.)<br>**Now, please repeat this after me: "Methodist Episcopal."**<br><u>No repetitions of the phrases are permitted</u>.		1 point for correct repetition<br>1 point for correct repetition	2	
9. **Who is the President of the United States right now?** (Pause for a response.)<br>**Who is the Vice-President?**<br>Both first and last names must be correct. If only the last name is given, probe for the full name.		1 point for current president's full name<br>1 point for current vice-president's full name	2	
10. **With your finger, tap five times on the part of the phone you speak into.**<br>If the TICS is being administered in person, the examinee should be asked to tap on the table rather than on a telephone receiver.		2 points if five taps are clearly heard<br>1 point if either more than or fewer than 5 taps are heard<br>0 points if no taps are heard.	2	
11. **I am going to say a word and I want you to give me its opposite. For example, if I said "hot," you would say "cold."**<br>**What is the opposite of "west"?** (Pause for a response.)<br>**What is the opposite of "generous"?**		1 point for "east"<br>1 point for "cheap," "stingy," "tight," "selfish," "greedy," "mean," "meager," or other correct antonym	2	

TICS Total score
Total possible points = 41
