# Supplementary material for: Delirium After Mechanical Ventilation in Intensive Care Units: The Cognitive and Psychosocial Assessment (CAPA) Study Protocol
Source: JMIR Res Protoc. 2017 Feb 28;6(2):e31. doi: 10.2196/resprot.6660 (PMC5426842; doi:10.2196/resprot.6660)
Supplement: Multimedia Appendix 3 [file resprot_v6i2e31_app3.pdf]

# IMPACT OF EVENT SCALE- REVISED

**INSTRUCTIONS:** Below is a list of difficulties people sometimes have after stressful life events. Please read each item, and then indicate how each situation has been for you **DURING THE PAST SEVEN DAYS** with respect to your experience while being treated in intensive care. How much were you distressed or bothered by these difficulties?

| | | | | |
|---|---|---|---|---|
| Not at all = 0 | A little bit = 1 | Moderately = 2 | Quite a bit = 3 | Extremely = 4 |

1. Any reminder brought back feelings about it.
2. I had trouble staying asleep.
3. Other things kept making me think about it.
4. I felt irritable and angry.
5. I avoided letting myself get upset when I thought about it or was reminded of it.
6. I thought about it when I didn't mean to.
7. I felt as if it hadn’t happened or wasn’t real.
8. I stayed away from reminders of it.
9. Pictures about it popped into my mind.
10. I was jumpy and easily startled.
11. I tried not to think about it.
12. I was aware that I still had a lot of feelings about it, but I didn't deal with them.
13. My feelings about it were kind of numb.
14. I found myself acting or feeling like I was back at that time.
15. I had trouble falling asleep.
16. I had waves of strong feelings about it.
17. I tried to remove it from my memory.
18. I had trouble concentrating.
19. Reminders of it caused me to have physical reactions, such as sweating, trouble breathing, nausea, or a pounding heart.
20. I had dreams about it.
21. I felt watchful and on-guard.
22. I tried not to talk about it.

---

Patient ID:_________________ Time-point:_________________

Date:_________________
