# Supplementary material for: Delirium After Mechanical Ventilation in Intensive Care Units: The Cognitive and Psychosocial Assessment (CAPA) Study Protocol
Source: JMIR Res Protoc. 2017 Feb 28;6(2):e31. doi: 10.2196/resprot.6660 (PMC5426842; doi:10.2196/resprot.6660)
Supplement: Multimedia Appendix 4 [file resprot_v6i2e31_app4.pdf]

Short Form of the Informant Questionnaire on Cognitive Decline in the Elderly (Short IQCODE)

Now we want you to remember what your friend or relative were like 5 years ago and to compare it with what he/she is like these days. Below are situations where this person has to use his/her memory or intelligence and we want you to indicate whether this has improved, stayed the same or got worse in that situation over the past 5 years. Note the importance of comparing his/her present performance with 5 years ago. So if 5 years ago this person always forgot where he/she had left things, and he/she still does, then this would be considered "Hasn't changed much". Please indicate the changes you have observed by circling the appropriate answer.

Compared with 5 years ago how is this person at:

	1	2	3	4	5
1. Remembering things about family and friends e.g. occupations, birthdays, addresses	Much improved	A bit improved	Not much change	A bit worse	Much worse
2. Remembering things that have happened recently	Much improved	A bit improved	Not much change	A bit worse	Much worse
3. Recalling conversations a few days later	Much improved	A bit improved	Not much change	A bit worse	Much worse
4. Remembering his/her address and telephone number	Much improved	A bit improved	Not much change	A bit worse	Much worse
5. Remembering what day and month it is	Much improved	A bit improved	Not much change	A bit worse	Much worse
6. Remembering where things are usually kept	Much improved	A bit improved	Not much change	A bit worse	Much worse
7. Remembering where to find things which have been put in a different place from usual	Much improved	A bit improved	Not much change	A bit worse	Much worse
8. Knowing how to work familiar machines around the house	Much improved	A bit improved	Not much change	A bit worse	Much worse

9. Learning to use a new gadget or machine around the house	Much improved	A bit improved	Not much change	A bit worse	Much worse
10. Learning new things in general	Much improved	A bit improved	Not much change	A bit worse	Much worse
11. Following a story in a book or on TV	Much improved	A bit improved	Not much change	A bit worse	Much worse
12. Making decisions on everyday matters	Much improved	A bit improved	Not much change	A bit worse	Much worse
13. Handling money for shopping	Much improved	A bit improved	Not much change	A bit worse	Much worse
14. Handling financial matters e.g. the pension, dealing with the bank	Much improved	A bit improved	Not much change	A bit worse	Much worse
15. Handling other everyday arithmetic problems e.g. knowing how much food to buy, knowing how long between visits from family or friends	Much improved	A bit improved	Not much change	A bit worse	Much worse
16. Using his/her intelligence to understand what's going on and to reason things through	Much improved	A bit improved	Not much change	A bit worse	Much worse

Patient ID:_________________ Time-point:_________________

Date:_________________
