# Supplementary material for: Delirium After Mechanical Ventilation in Intensive Care Units: The Cognitive and Psychosocial Assessment (CAPA) Study Protocol
Source: JMIR Res Protoc. 2017 Feb 28;6(2):e31. doi: 10.2196/resprot.6660 (PMC5426842; doi:10.2196/resprot.6660)
Supplement: Multimedia Appendix 5 [file resprot_v6i2e31_app5.pdf]

PAR
Creating Connections. Changing Lives.

16204 N. FLORIDA AVENUE • LUTZ, FLORIDA 33549
Telephone: 813.968.3003 • Fax: 813.968.2598 • Web: www.parinc.com

Sent Via Email: daniella.bulic@hotmail.com

June 11, 2014

Daniella Bulic
University of New South Wales
POWCS, Faculty of Medicine
Level 1, Dickinson Building
High Street
Randwick, Sydney 2031
Australia

Dear Ms. Bulic:

In response to your recent request, permission is hereby granted to you to include the four (4) approved sample items from the Mini-Mental State Examination (MMSE) Test Form and up to a total of three (3) sample items from the Telephone Interview for Cognitive Status (TICS) Record Form in your PhD Thesis titled, *Cognitive and Psychosocial Assessment After Mechanical Ventilation in Intensive Care: Does an Experience of Delirium Make a Difference?: CAPA Study.*

The four (4) approved sample items for the MMSE are provided to you in Appendix A of this Permission Agreement. No other sample items will be allowed to be published from the MMSE Test Form. **PAR will not grant permission to publish the entire MMSE Test Form in any publication.**

This Agreement is subject to the following restrictions:

(1) Any and all materials used will contain the following credit line:

MMSE:

"Reproduced by special permission of the Publisher, Psychological Assessment Resources, Inc., 16204 North Florida Avenue, Lutz, Florida 33549, from the Mini Mental State Examination, by Marshal Folstein and Susan Folstein, Copyright 1975, 1998, 2001 by Mini Mental LLC, Inc. Published 2001 by Psychological Assessment Resources, Inc. Further reproduction is prohibited without permission of PAR, Inc. The MMSE can be purchased from PAR, Inc. by calling (813) 968-3003."

PAR
Creating Connections. Changing Lives.

16204 N. FLORIDA AVENUE • LUTZ, FLORIDA 33549
Telephone: 813.968.3003 • Fax: 813.968.2598 • Web: www.parinc.com

TICS:

"Reproduced by special permission of the Publisher, Psychological Assessment Resources, Inc., 16204 North Florida Avenue, Lutz, Florida 33549 from the Telephone Interview for Cognitive Status by Jason Brandt, Ph.D. and Marshal F. Folstein, M.D., Copyright 1987, 2003. Further reproduction is prohibited without permission of the Publisher."

(2) None of the material may be sold, given away, or used for purposes other than those described above.

(3) Payment of a permission fee will be waived.

(4) One copy of any of the material reproduced will be sent to PAR to indicate that the proper credit line has been used.

TWO COPIES of this Permission Agreement should be signed and returned to me to indicate your agreement with the above restrictions. I will then sign it for PAR and return a fully executed copy to you for your records.

Sincerely,

Vicki M. McFadden
Permissions Specialist
vmark@parinc.com
1-800-331-8378 (phone)
1-800-727-9329 (fax)

ACCEPTED AND AGREED:

BY: [signature]
DANIELLA BULIC

DATE: 12/06/2014

ACCEPTED AND AGREED:

BY: [signature]
VICKI M. MCFADDEN

DATE: June 16, 2014

PAR
Creating Connections. Changing Lives.
16204 N. FLORIDA AVENUE • LUTZ, FLORIDA 33549
Telephone: 813.968.3003 • Fax: 813.968.2598 • Web: www.parinc.com

Appendix A

MMSE Sample Items

Orientation to Time

"What is the date?"

Registration

"Listen carefully. I am going to say three words. You say them back after I stop. Ready? Here they are...

APPLE (pause), PENNY (pause), TABLE (pause). Now repeat those words back to me." [Repeat up to 5 times, but score only the first trial.]

Naming

"What is this?" [Point to a pencil or pen.]

Reading

"Please read this and do what it says."

[Show examinee the words on the stimulus form.]

CLOSE YOUR EYES

"Reproduced by special permission of the Publisher, Psychological Assessment Resources, Inc., 16204 North Florida Avenue, Lutz, Florida 33549, from the Mini Mental State Examination, by Marshal Folstein and Susan Folstein, Copyright 1975, 1998, 2001 by Mini Mental LLC, Inc. Published 2001 by Psychological Assessment Resources, Inc. Further reproduction is prohibited without permission of PAR, Inc. The MMSE can be purchased from PAR, Inc. by calling (813) 968-3003."
